# Oxidative Stress Response in Adipose Tissue-Derived Mesenchymal Stem/Stromal Cells

**DOI:** 10.3390/ijms232113435

**Published:** 2022-11-03

**Authors:** Tawakalitu Okikiola Waheed, Olga Hahn, Kaarthik Sridharan, Caroline Mörke, Günter Kamp, Kirsten Peters

**Affiliations:** 1 Department of Cell Biology, University Medical Centre Rostock, Schillingallee 69, 18057 Rostock, Germany; 2 AMP-Lab GmbH, Mendelstr. 11, 48149 Münster, Germany

**Keywords:** reactive oxygen species, oxidative stress, mesenchymal stem cells, hydrogen peroxide, glucose oxidase, wound healing

## Abstract

Reactive oxygen species (ROS) can irreversibly damage biological molecules, a process known as oxidative stress. Elevated ROS levels are associated with immune cell activation. Sustained immune system activation can affect many different cells in the environment. One cell type that has been detected in almost all tissues of the body is mesenchymal stem/stromal cells (MSC). MSC possess proliferation and differentiation potential, thus facilitating regeneration processes. However, the regenerative capacity of MSC might be impaired by oxidative stress, and the effects of long-term oxidative stress on MSC functions are sparsely described. The examination of oxidative stress is often performed by exposure to H_2_O_2_. Since H_2_O_2_ is rapidly degraded, we additionally exposed the cell cultures to glucose oxidase (GOx), resulting in sustained exposure to H_2_O_2_. Using these model systems, we have focused on the effects of short- and long-term oxidative stress on viability, migration, differentiation, and signaling. All cellular functions examined were affected by the applied oxidative stress. The differences that occur between pulsed and sustained oxidative stress indicated higher oxidative stress in MSC upon direct H_2_O_2_ exposure, whereas the GOx-induced prolonged exposure to H_2_O_2_ seems to allow for better cellular adaptation. The mechanisms underlying these different responses are currently unknown.

## 1. Introduction

Reactive oxygen species (ROS) are highly reactive compounds derived from oxygen and formed by the capture of electrons by an oxygen atom [1]. ROS are by-products of normal metabolism and play a role in cell signaling and homeostasis by regulating cellular proliferation, differentiation, and survival [2]. However, ROS can also cause irreversible damage to biological molecules (i.e., DNA, proteins, lipids) [3,4]. To protect biological macromolecules from oxidative stress, mammalian cells have evolved a sophisticated antioxidant defense system that includes molecules (e.g., glutathione (GSH), ascorbic acid), enzymes (e.g., catalase, GSH peroxidase (GPX), superoxide dismutase (SOD)) [5]. The condition in which there is an imbalance between the levels of ROS or reactive nitrogen species (RNS) and the antioxidant molecules, as well as enzymes, is termed oxidative stress [6,7]. The three major physiological significance ROS molecules are hydroxyl radical (^•^OH), superoxide anion (O_2_^•−^), and hydrogen peroxide (H_2_O_2_) [6]. A decrease in antioxidant levels with increased ROS accumulation can lead to cellular dysfunction or damage and may result in cell death [8]. 

Elevated ROS levels are often connected to the activation of immune cells (e.g., macrophages and neutrophils) releasing ROS, which is known as an oxidative burst. As part of innate immunity, this process is essential for the degradation of internalized pathogens (e.g., bacteria) [9]. However, this process also involves risks. In wound healing, for example, the double-edged nature of ROS release by immune cells becomes apparent. Whereas slightly elevated ROS levels exert an antimicrobial effect by activating pro-inflammatory signaling pathways and immune cells, excessive ROS levels lead to destructive effects in the wound, which perpetuate inflammation and can also chronify the wound healing process leading to wound healing disorder [10,11]. This chronification can affect many different cells in the afflicted tissue regions, causing fibrotic reactions compromising tissue functions, such as in fibrotic lung or diabetic wounds [12,13]. One cell type that has been identified in almost all tissues of the body, including subcutaneous tissue, is the mesenchymal stem/stromal cells (MSC) [14,15]. 

MSC have a high proliferation and differentiation potential. These properties allow them to renew the fraction of mesenchymal progenitor cells or to differentiate into tissue-specific cell types [16,17]. Thus, these properties contribute to the maintenance and regeneration of supportive and connective tissue, such as bone, cartilage, tendon, or adipose tissue [15,18]. MSC are considered to facilitate regeneration processes by exerting anti-inflammatory and immunomodulatory effects through the release of cytokines and other soluble factors, in addition to the aforementioned proliferative and differentiation properties [19,20]. Therefore, the cell therapeutic use of MSC is of great interest in many respects, which is also reflected in the large number of clinical studies involving MSC [21]. However, the success of MSC in cell therapy depends on various important factors. These include nutrient supply, accessibility of growth factors, and oxidative stress [22,23]. The regenerative capacity of MSC might be impaired by an unfavorable coincidence of the levels of these three factors, as mentioned earlier. 

The effects of oxidative stress are often illustrated in vitro by exposure to the oxidative stressor H_2_O_2_ [24,25,26]. H_2_O_2_ is one of the most important compounds for the study of ROS signaling due to its physicochemical properties, which are relatively low reactivity and the ability to diffuse through membranes [27]. A number of molecules that respond to H_2_O_2_-induced oxidative stress include superoxide dismutase (SOD), catalase, and glutathione peroxidase (GPx) [28]. However, many questions about the effects of oxidative stress in MSC, such as the effects of long-term exposure to H_2_O_2_ or ROS, are still unclear. The effects of long-term ROS exposure are of particular interest as persistent ROS production has been reported in the chronification of inflammatory processes [29,30]. ROS initiates signaling pathways that activate several transcription factors, such as nuclear factor kappa B (NF-ҡB), by initiating its release from its inhibitor IҡB and facilitating its translocation into the nucleus. Thereafter, NF-ҡB induces several genes responsible for the activation of pro-inflammatory cytokines and chemokines [3].

Therefore, this study focused on understanding the effects of short- and long-term oxidative stress induced by H_2_O_2_ exposure on MSC from adipose tissue (adipose tissue-derived MSC, adMSC) in vitro. Not only were the adMSC treated with H_2_O_2_, but also with glucose oxidase (GOx), an enzyme that produces H_2_O_2_ by glucose oxidation [31]. In this way, sustained production of H_2_O_2_ was induced in the culture medium. Thus, we induced two different characteristics of H_2_O_2_ exposure: the first rather pulsatile, the second rather persistent. 

In this study, we quantified the amount of H_2_O_2_ and ROS present or generated after treatments with these oxidative stressors and investigated their effects on the proliferation, viability, migration, and differentiation of adMSC. In addition, the underlying signaling processes were investigated. These studied aspects are important influencing variables of the regeneration and wound healing process [32,33]. Understanding the influence of long-term oxidative stress on MSC in terms of the variables described above might also provide a basis for improving the therapeutic efficacy of MSC in the wound healing process.

## 2. Results

### 2.1. Quantification of Extracellular H_2_O_2_ and Intracellular Reactive Oxygen Species (ROS)

**Figure 1:**
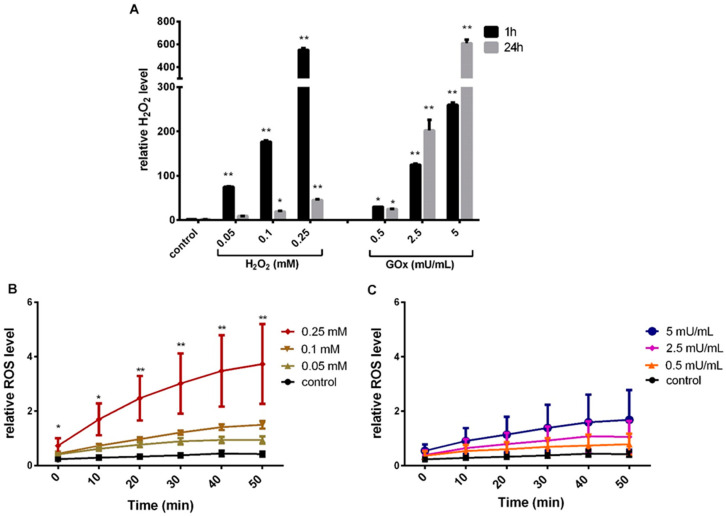
Quantification of H_2_O_2_ in the cell culture medium and analysis of the formation of intracellular ROS. (**A**) Quantification of H_2_O_2_ in the cell culture medium treated with different concentrations of H_2_O_2_ and GOx for 1 and 24 h. (**B**) Intracellular ROS levels were quantified after treatment with different concentrations of H_2_O_2_ and (**C**) GOx for 50 min (data presented as bar graphs for H_2_O_2_ concentration measured from the fluorescent intensity with Amplex UltraRed, and ROS measured from relative fluorescence intensity normalized to cell count with CM-H_2_DCFDA ROS indicator, n = 5, mean and standard deviation of the mean, significance compared to the respective control, two-way ANOVA with Dunnett’s multiple comparison test, * *p* < 0.05, ** *p* < 0.001).

To determine the concentration of H_2_O_2_ in the cell culture medium, the Amplex UltraRed assay was performed. This measurement allows us to assess the amount of H_2_O_2_ added or produced by GOx and the scavenging capacity of the cell culture medium. For this purpose, different concentrations of H_2_O_2_ and GOx (H_2_O_2_: 0.01, 0.05, 0.1 and 0.25 mM, and GOX: 0.1, 0.5, 1, 2.5, and 5 mU/mL) were added to the cell culture medium (Dulbecco’s Modified Eagle’s Medium/DMEM containing 10% fetal calf serum/FCS, 1% penicillin/streptomycin/P/S, 0.4% GlutaMax^TM^ without pyruvate). The quantification of H_2_O_2_ was carried out for 48 h at different time points. Directly added H_2_O_2_ in the cell culture medium was almost completely degraded over a period of 48 h (at all H_2_O_2_ concentrations except 0.25 mM, where approximately 5% of the initially applied amount remained). After 24 h of exposure, a maximum of 10% of the initially applied amount was detectable in each case (Figure 1A and Figure S1A). In contrast, GOx exposure led to an enzyme activity-dependent significant increase in the amount of H_2_O_2_ in the cell culture medium, which largely remained over the observation period of 48 h (Figure 1A and Figure S1B). 

To determine the effect of H_2_O_2_ exposure on the amount of intracellular ROS, cell cultures were incubated with the redox-sensitive substance 2′-7′-dichlorofluorescein diacetate (CM-H2DCFDA) and thereafter exposed to H_2_O_2_ or GOx, and the resulting fluorescence was quantified. The intracellular ROS amount increased dose- and time-dependently (significant at 0.25 mM H_2_O_2_) and was nearly saturated after 50 min in H_2_O_2-_treated adMSC (Figure 1B and Figure S1C). After GOx exposure, a time- and concentration-dependent increase in the amount of ROS in the adMSC was observed (Figure 1C and Figure S1D). However, GOx-induced ROS accumulation was not as high as in adMSC treated with H_2_O_2_. The highest amount of GOx induces less than half of the ROS compared to the highest amount of H_2_O_2_ after 50 min, in accordance with the higher amount of H_2_O_2_ measured after 1 h for the direct H_2_O_2_ addition compared to the H_2_O_2_ production induced by GOx.

### 2.2. Quantification of Cell Number and Metabolic Activity

**Figure 2:**
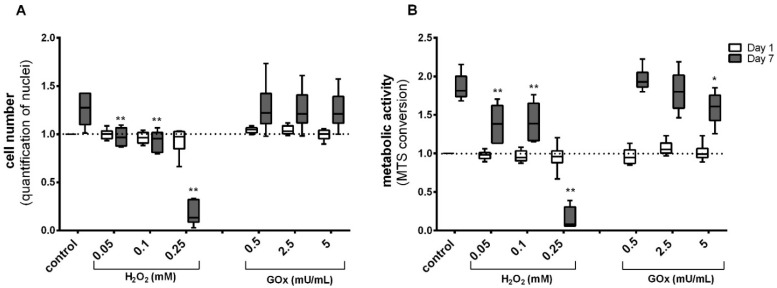
The effect of H_2_O_2_ and GOx on (**A**) cell numbers and (**B**) metabolic activity after day 1 and day 7 of repeated treatment; ((**A**) cell number analyzed by quantification of nuclei upon staining with Hoechst H33342, (**B**) metabolic activity quantified by MTS conversion assay). The data set was normalized to the control on day 1 after the treatment; (n = 6, significance compared to the control group on the respective days of the treatment, two-way ANOVA with Dunnett’s multiple comparison test, * *p* < 0.05, ** *p* < 0.001).

The toxicity threshold of H_2_O_2_ and GOx was evaluated in adMSC for up to 7 days. Therefore, the cell numbers and the metabolic activity (MTS conversion assay) were determined after the exposure to H_2_O_2_ and GOx in different concentrations (as stated above). The H_2_O_2_ and GOx treatment took place every second day. This results in different treatment frequencies depending on the respective analysis time point. After 1 day of treatment, no significant change in adMSC number (Figure 2A) and metabolic activity (Figure 2B) was observed. Also, no apparent differences could be detected on the 3rd day of exposure (i.e., after two treatments) (Figure S2). However, after 7 days of exposure with three repeated treatments, a threshold decrease in cell number was observed at higher H_2_O_2_ concentrations, which was significant (*p* < 0.001) from 0.05 mM H_2_O_2_ (Figure 2A and Figure S2A). A similar effect was seen for the metabolic activity, where a significant reduction occurred after 7 days with H_2_O_2_ concentration from 0.05 mM (Figure 2B and Figure S2B). A clear cytotoxic effect was visible in adMSC treated with the highest H_2_O_2_ concentration of 0.25 mM. In contrast, the treatment with all concentrations of GOx did not induce a cytotoxic effect. However, a significant decrease in metabolic activity was observed in the cells treated with 5 mU GOx/mL. For this reason, only non-toxic concentrations of H_2_O_2_ and GOx were used for the subsequent investigations.

### 2.3. Profiling of Cell Stress Protein Expression

**Figure 3:**
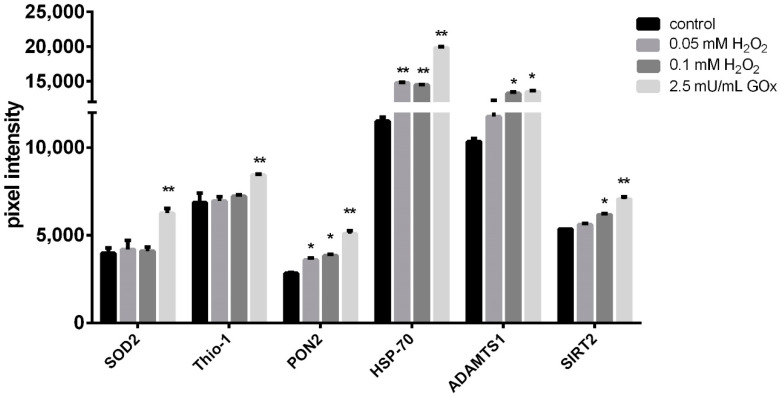
Analysis of human cell stress proteins. adMSC were treated with 0.05 and 0.1 mM H_2_O_2,_ or 2.5 mU/mL GOx, respectively. adMSC lysates after 24 h of treatment were analyzed with ‘Proteome Profiler Human Cell Stress Array Kit’ (pixel intensity of the protein spots quantified by densitometry analysis using the Image Lab 3.0.1 software; n = 5, significance compared to the control, 2-way ANOVA with Dunnett’s multiple comparison test, * *p* < 0.05, ** *p* < 0.001).

To investigate the extent of stress exerted by H_2_O_2_ exposure on adMSC, a ‘Proteome Profiler Human Cell Stress Array Kit’ was used. This kit allows the simultaneous analysis of 26 cell stress-related proteins (Table S1). For this purpose, lysates of adMSC after 24- and 48-h treatment with 0.05, 0.1 mM H_2_O_2_, and 2.5 mU/mL GOx, were prepared and subjected to membrane/antibody-based analysis, and the blots were subsequently quantified (Figure S3A,B). The protein levels of six of these cell stress-associated proteins (i.e., 23% of the proteins analyzed) were differentially regulated in response to H_2_O_2_ and GOx exposure, respectively. The following proteins were elevated in lysates from adMSC treated for 24 h with H_2_O_2_ and GOx compared to the control: superoxide dismutase 2 (SOD2), thioredoxin-1 (Thio-1), paraoxonase 2 (PON2), heat shock protein-70 (HSP-70), ADAM metallopeptidase 1 (ADAMTS1), and sirtuin 2 (SIRT2) (Figure 3). The most significant effects were observed in adMSC treated with 2.5 mU GOx/mL. In contrast, lysates obtained from adMSC treated for 48 h with the oxidative stressors showed a significant reduction in expression of these same stress proteins in a dose-dependent manner compared to the control (Figure S3C).

### 2.4. Oxidative Stress and Inflammatory Signaling

**Figure 4:**
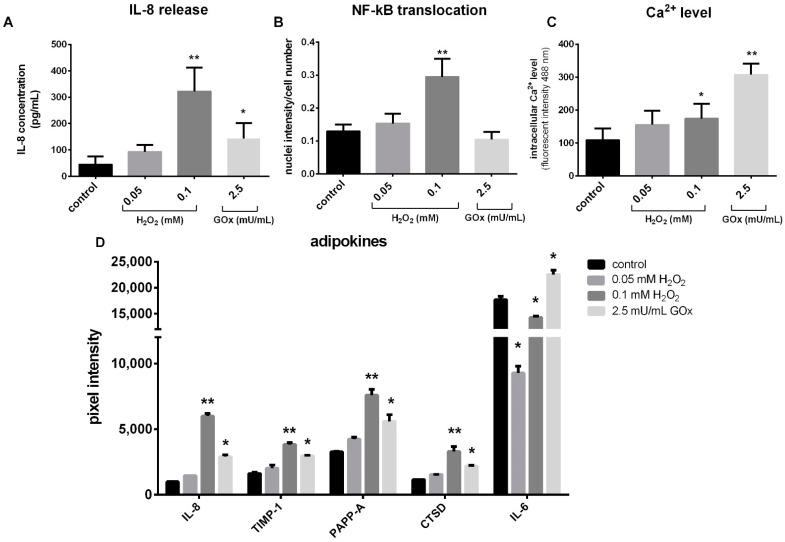
Inflammation-related signaling upon treatment of adMSC with 0.05, 0.1 mM H_2_O_2,_ and 2.5 mU/mL GOx. (**A**) Quantification of IL-8 releases into the supernatant (analysis by enzyme-linked immunosorbent assay, ELISA). (**B**) Quantification of nuclear translocation of NF-ҡB normalized to cell number (analysis of nuclei fluorescence by Athena software). (**C**) Intracellular Ca^2+^ level detection with the fluo-3/AM calcium indicator (analysis by flow cytometry). (**D**) Depiction of differentially released adipokines with ‘Human Adipokine Array Kit’ (pixel intensities were quantified by densitometry using the Image Lab 3.0.1 software, n = 5, significance compared to the control, two-way ANOVA with Dunnett’s multiple comparison test, * *p* < 0.05, ** *p* < 0.001).

The influence of oxidative stress on inflammatory reactions of adMSC was investigated by the quantification of the proinflammatory cytokine Interleukin-8 (IL-8). adMSC treated with 0.05, 0.1 mM H_2_O_2_, and 2.5 mU/mL of GOx for 48 h showed an increased release of IL-8 in the cell culture supernatant. The most significant effect was observed in 0.1 mM H_2_O_2_-treated adMSC, where 322 pg IL-8/mL was detected compared to 46 pg/mL in the untreated control cultures (Figure 4A). To further investigate the involvement of oxidative stressors in proinflammatory signaling, the nuclear translocation of NF-ҡB, an inducer of various inflammatory genes was analyzed. A significant increase in nuclear translocation of NF-ҡB was observed in adMSC treated with 0.1 mM H_2_O_2_ for 24 h (Figure 4B). The lower tested H_2_O_2_ concentration (0.05 mM) and 2.5 mU/mL of GOx did not induce marked differences in nuclear translocation at the two tested time points of 8 and 24 h.

Furthermore, the effect of H_2_O_2_ exposure on the intracellular calcium (Ca^2+^) level was investigated. The Ca^2+^ concentration was measured by flow cytometry after the treatment of adMSC with different concentrations of H_2_O_2_ and GOx for 24 h (Figure S4A). Intracellular Ca^2+^ levels were dose-dependent and starting at a concentration of 0.1 mM H_2_O_2_, and 2.5 mU/mL significantly increased (Figure 4C). 

In addition, the supernatants of adMSC treated with 0.05, 0.1 mM H_2_O_2_, and 2.5 mU/mL GOx were analyzed for adipogenesis and obesity-related factors (adipokines) in response to oxidative stress using a ‘Human Adipokine Array kit’ (Figure S4B). This array allows the simultaneous quantification of 58 obesity-related factors (Table S2). The densitometric evaluation of the membranes revealed that the ‘classic’ adipokines, such as leptin or adiponectin, were not regulated by H_2_O_2_ or GOx. However, the factors that mainly influence the inflammatory processes, such as IL-6, IL-8, and tissue inhibitor of metalloproteinase 1 (TIMP-1), were differentially regulated by exposure to the oxidative stressors (Figure 4D). Thus, the release pattern of IL-8, TIMP-1, metalloproteinases-related protein pappalysin-1 (PAPP-A), and the lysosomal protease cathepsin D (CTSD) was similar in that the two H_2_O_2_ concentrations resulted in a concentration-dependent increase in releases, and exposure to 2.5 mU/mL caused a milder increase compared to the higher H_2_O_2_ concentration. Only the release pattern of IL-6 differs; here, GOx exposure causes a significant increase in the release from adMSC, whereas H_2_O_2_ exposure causes a significant decrease (Figure 4D). 

### 2.5. adMSC Migration after Exposure to H_2_O_2_ and GOx

**Figure 5:**
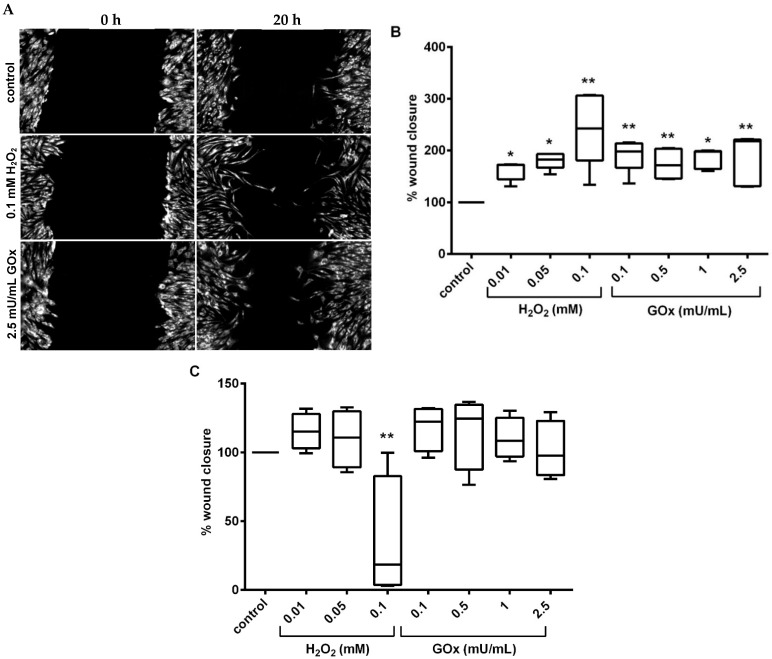
Migratory capacity of adMSC tested by the scratch assay. (**A**) Fluorescent depiction of adMSC preconditioned with H_2_O_2_ and GOx for 24 h in the scratch area (referred to as setting 1: cell staining was with cell tracking red dye, scale bar: 50 µm), and imaging at an initial time point, 0 h, and 20 h after scratching. (**B**) Quantification of wound closure after cells were preconditioned 24 h with H_2_O_2_ and GOx before scratch, and no incubation with H_2_O_2_ and GOx during migration (setting one); (**C**) adMSC preconditioned 2 h before scratch and exposed to H_2_O_2_ and GOx during migration (setting two). % wound closure normalized to the control; (n = 5, significance compared to the control, two-way ANOVA with Dunnett’s multiple comparison test, * *p* < 0.05, ** *p* < 0.001).

To evaluate the effect of oxidative stress on the migratory capacity of adMSC, we performed a so-called wound healing assay (also known as scratch assay). This test was performed under two different settings. First setting: the cells were treated with the oxidative stressors (H_2_O_2_: 0.01, 0.05, and 0.1 mM and GOx: 0.1, 0.5, 1, and 2.5 mU/mL) for 24 h before scratch and were not further treated with the oxidative stressors after scratching. Second setting: cells were both pre-treated with the oxidative stressors and further treated with the oxidative stressors after scratching. In setting one, the fluorescent depiction of the scratch area indicated an increased migratory capacity in all treated adMSC compared to the control, as more cells covered the scratch area (Figure 5A). Quantification of wound closure (in % of the scratch area) revealed that the first setting led to a significantly increased migratory activity of adMSC after pre-incubation with oxidative stressors and subsequent absence of oxidative stress during migration (Figure 5B). 

In contrast, the second setting, where cells were pre-treated with the oxidative stressors for 2 h and continued exposure to the oxidative stressors after scratching, does not significantly increase the migratory activity. Lower H_2_O_2_ concentrations or GOx activities tested led to a slight increase in the migration of adMSC. On the other hand, the adMSC migration was almost entirely suppressed by increasing the amount of H_2_O_2_ to 0.1 mM. This means that there is a high sensitivity of adMSC to the extent of oxidative stress. The GOx-induced H_2_O_2_ exposure did not lead to a change in adMSC migration at any time (Figure 5C).

### 2.6. Adipogenic Differentiation of adMSC under Oxidative Stress 

**Figure 6:**
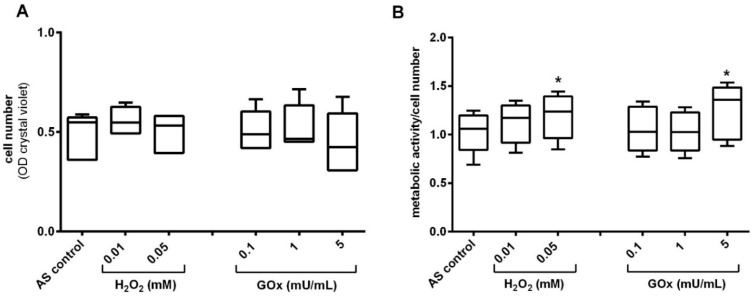

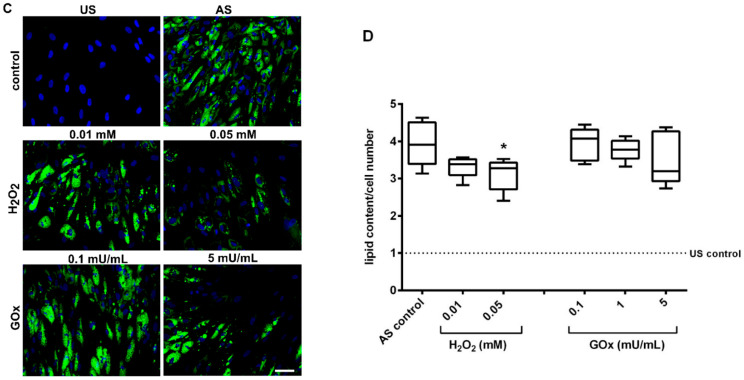
Adipogenic differentiation in oxidatively stressed adMSC after 14 days. (**A**) Quantification of relative cell number in adipogenic stimulated (AS) adMSC (quantified with crystal violet staining) and (**B**) metabolic activity (quantified by MTS conversion assay related to the cell amount). (**C**) Fluorescent depiction of accumulated lipid in unstimulated (US) control cultures, adipogenic stimulated control cultures, and adipogenically stimulated and oxidatively stressed adMSC treated with 0.01 and 0.05 mM H_2_O_2_, and 0.1 and 5 mU/mL GOx, respectively; (nuclei stained with Hoechst H33342, lipid accumulation stained with Bodipy, representative images, scale bar: 50 µm). (**D**) Quantification of fluorescence intensity of the lipid staining after 14 days of stimulation and repeated treatments with different concentrations of H_2_O_2_ and GOx (normalized values to unstimulated control; n = 5, significance compared to the stimulated control, two-way ANOVA with Dunnett’s multiple comparison test, * *p* < 0.05).

The adipogenic differentiation capacity of adMSC under oxidative stress was demonstrated by the detection and quantification of lipid accumulation after 14 days of adipogenic stimulation and repeated treatments with oxidative stressors. At the end of the 14-day adipogenic stimulation period, there were no H_2_O_2_- or GOx-induced changes in cell numbers compared to the untreated control (Figure 6A). Interestingly the metabolic activity (determined by MTS conversion assay) of H_2_O_2_/GOx treated and simultaneously adipogenically stimulated adMSC increased concentration-dependently (Figure 6B).

The depiction of lipid accumulation after 14 days revealed a clear adipogenic differentiation of adMSC, whereas non-adipogenic stimulated adMSC (unstimulated, US) were free of lipid accumulation (Figure 6C). Notably, upon H_2_O_2_ treatment, even the lowest concentration tested (0.01 mM) led to an apparent reduction in the number of lipids (Figure 6C). The quantification of the lipid accumulation also confirmed the reduction. Exposure to all oxidative stressors resulted in a concentration-dependent decrease in the number of lipids. However, this effect was more pronounced after H_2_O_2_ exposure than after exposure to GOx (Figure 6D).

### 2.7. Osteogenic Differentiation of adMSC under Oxidative Stress

**Figure 7:**
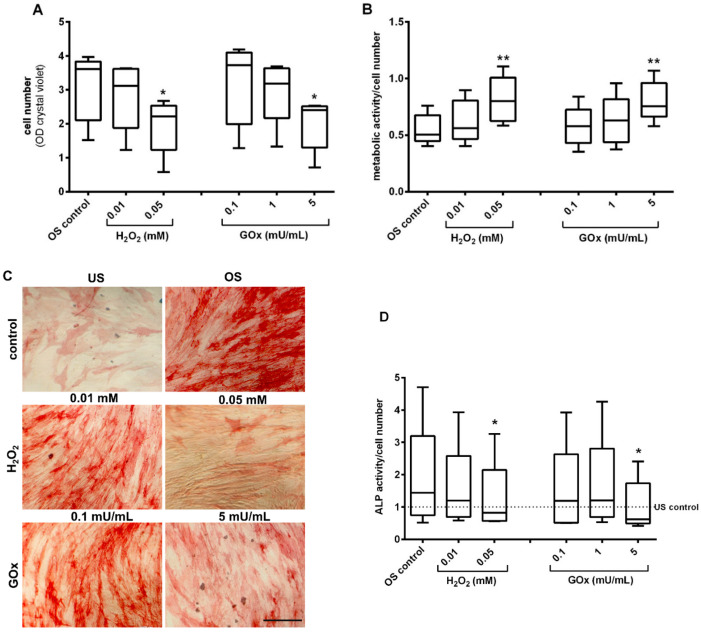
Osteogenic differentiation in oxidatively stressed adMSC after 21 days. (**A**) Quantification of relative cell number in osteogenic stimulated (OS) adMSC (quantified with crystal violet staining) and (**B**) metabolic activity (quantified by MTS conversion assay related to the cell amount). (**C**) Microscopic depiction of ALP staining in untreated and unstimulated (US) control, untreated and OS control, OS adMSC treated with 0.01 mM and 0.05 mM H_2_O_2_, 0.1 and 5 mU/mL GOx, respectively (representative images, scale bar: 100 µm). (**D**) Quantification of ALP activity by p-Nitrophenyl Phosphate (pNPP) after 21 days of osteogenic stimulation and repeated treatment with different concentrations of H_2_O_2_ and GOx (normalized values to unstimulated control; n = 5, significance compared to the stimulated control; two-way ANOVA with Dunnett’s multiple comparison test, * *p* < 0.05, ** *p* < 0.001).

Osteogenic differentiation capacity was investigated by the measurement of alkaline phosphatase (ALP) activity, a bone-specific enzyme, and an early marker for osteogenic stimulation. The relative cell amount was determined after 21 days of osteogenic stimulation and repeated H_2_O_2_-/GOx-treatments. A dose-dependent decrease in relative cell amount was observed in adMSC repeatedly treated with H_2_O_2_ and GOx compared to the osteogenically stimulated, non-oxidatively stressed control (significant at 0.05 mM H_2_O_2_ and 5 mU/mL GOx) (Figure 7A). In contrast, the metabolic activity increased significantly (*p* < 0.001) in osteogenically stimulated adMSC with increasing concentrations of H_2_O_2_ and GOx (Figure 7B). The detection of ALP activity showed only a few slightly ALP-positive cells in the unstimulated adMSC after 21 days of cultivation (Figure 7C). In contrast, the osteogenically stimulated cells were clearly ALP-positive after 21 days (Figure 7C). Osteogenically stimulated adMSC, repeatedly treated with H_2_O_2_ and GOx, indicated a concentration-dependent decrease in ALP activity. The result was supported by photometrical quantification of ALP activity (normalized to cell amount). Where the osteogenic stimulation and simultaneous treatment of adMSC with H_2_O_2_ and GOx reduce the ALP activity in a dose-dependent manner (significantly at 0.05 mM H_2_O_2_ and 5 mU/mL GOx with *p* < 0.05) (Figure 7D).

## 3. Discussion

ROS serve as an essential signaling molecule in the maintenance of cellular functions [3,34]. ROS are involved in coordinating effective tissue repair by regulating different cell types, including blood vessel formation. Given this important role of ROS, understanding the detailed background of ROS signaling is a promising route for improving tissue repair and potentially influencing processes by balancing the ROS composition [30]. In this context, ROS could also have an impact on the clinical application of MSC [35]. In contrast, excessive ROS production induces oxidative stress in the cells, which has been associated with the progression of many diseases [4,36]. Although the effects of oxidative stress have been studied widely, many questions remain unanswered due to the complex interrelationships of many influencing factors and heterogeneous reported results. We have focused our investigations on MSC because they are an important cell type in tissue regeneration and hold promise for cell therapy in various diseases. The capacity to maintain MSC properties depends on the balance of complex signals in their microenvironment, partly regulated by ROS [37]. For example, MSC engraftment in the case of chronic wounds has been proposed to be an efficient method of promoting wound healing [38,39]. However, their therapeutic efficacy in the injured tissue is limited because of their low survival rate and poor engraftment efficiency due to oxidative stress [40]. Therefore, understanding the contributions of oxidative stress in the regenerative capacity and the survival of MSC in the injured tissue microenvironment is essential to promote MSC engraftment and enhance tissue repair. 

### 3.1. Oxidative Stress Signaling in MSC

A number of in vitro studies have investigated the effects of H_2_O_2_ on oxidative stress response in MSC [41,42,43,44]_._ The stability of H_2_O_2_ in biological environments is fundamentally limited, and antioxidant substances in biological environments (e.g., vitamins, carotenoids, flavonoids, and antioxidant enzymes) shorten the shelf life of H_2_O_2_ [5]. This basically results in pulsed exposure when using H_2_O_2_ as a model substance for oxidative stress. For this reason, we have additionally chosen exposure with GOx for our in vitro model. GOx produces H_2_O_2_ in a two-step redox reaction, and the concentration of the end product (H_2_O_2_) depends on enzyme activity and substrate (glucose) availability [31]. Treatment with GOx has been evaluated by extensive studies on the wound-healing ability of medicinal honey. In the wound-healing environment, GOx could exert its antimicrobial activity through the production of H_2_O_2_ [45].

Our results confirm that the H_2_O_2_ concentration in the medium after direct exposure to H_2_O_2_ is significantly reduced over 24 h. In contrast, the H_2_O_2_ concentration after GOx exposure in the medium is relatively stable over the period of 48 h. Therefore, we refer to direct H_2_O_2_ exposure as pulsed H_2_O_2_ exposure in the following discussion, whereas we refer to GOx treatment as sustained H_2_O_2_ exposure. It is important to note that the resulting maximal amounts of H_2_O_2_ detected were almost the same for the direct H_2_O_2_ treatment or with GOx activities we investigated. However, adMSC direct exposure to H_2_O_2_ leads to a significantly higher intracellular ROS accumulation compared to exposure to GOx (H_2_O_2_ induces more than twice as high ROS quantities). We assumed that cellular response to the exposure of both oxidative stress types could differ.

Cells can activate several signaling pathways in response to the elevation of ROS production [46]. These signaling pathways are essential for cell redox regulation or controlled ROS generation [47]. Activation of these signaling pathways leads to the upregulation of several stress proteins to maintain cellular homeostasis. The main systems for scavenging ROS in cells are antioxidant enzymes [5]. Our experiments showed that exposure of adMSC to both H_2_O_2_ and GOx for 24 h led to an elevation of stress proteins, PON2, ADAMTS1, Sirt2, and HSP-70, also antioxidant proteins, SOD2 and Thio-1. We observed a more prominent elevation of these proteins in adMSC exposed to GOx (2.5 mU/mL). The increase in these proteins was confirmed for their cytoprotective effect. For example, HSP-70 works together with antioxidant systems to inhibit the ROS effect and impair the apoptotic mechanism [48]. Elevations in antioxidant enzymes enhance adMSC resistance to oxidative damage. The lower cytotoxic effect of GOx-induced H_2_O_2_ exposure compared to pulsed H_2_O_2_ exposure that we have shown could be due to the higher increase in antioxidant factors in GOx. However, exposure of adMSC to oxidative stressors for 48 h led to a decrease in the expression of the mentioned antioxidant factors. This indicates that prolonged ROS generation might induce exhaustion of antioxidant enzymes. Moreover, disturbed redox equilibrium between ROS and antioxidants confers cellular dysfunction and progresses to oxidative damage [6,7].

Furthermore, ROS initiate several signaling processes, including the expression of genes responsible for survival through the activation of transcription factors, like NF-ҡB and hypoxia-inducible factor-1*α* (HIF-1*α*) [3]. ROS initiate the activation of protein kinase, thus the release of NF-ҡB from its inhibitor IҡB, which is then translocated into the nucleus, where it induces the expression of several genes responsible for the activation of pro-inflammatory cytokines and chemokines. Once triggered, these cytokines and chemokines bind to their respective receptors to regulate redox-sensitive pathways, resulting in numerous cellular responses [3]. Our result showed an elevation of NF-ҡB with the 24 h treatment of adMSC with 0.1 mM H_2_O_2_, and in turn, pro-inflammatory cytokines IL-6 and IL-8 confirmed ROS facilitated the production of these cytokines. GOx-induced H_2_O_2_ exposure in adMSC instigates lower inflammatory responses.

Several studies have shown that intracellular Ca^2+^ regulates the generation and degradation of ROS, thereby maintaining the redox state in the cell [46]. Therefore, we investigated the effects of oxidative stress in adMSC on intracellular Ca^2+^ levels. Exposure of adMSC to oxidative stressors increased the cytosolic Ca^2+^ levels dose-dependently. However, we observed a more pronounced increase in cytosolic Ca^2+^ in adMSC exposed to GOx-induced H_2_O_2_ than direct H_2_O_2_ exposure. An increase in intracellular Ca^2+^ has also been described upon H_2_O_2_ treatment in human endometrial stem cells [49]. The interaction between ROS and Ca^2+^ is bidirectional; increased Ca^2+^ concentrations activate ROS-generating enzymes and free radical formation, leading to increased ROS production [50]. There is increasing evidence that this cross-talk plays an essential role in ROS influence in the pathogenesis of several diseases.

### 3.2. Migration of MSC under Oxidative Stress

In a disease state with increased inflammation and oxidative stress, the migration of MSC is another important property required for their regenerative activity [41]. However, little is known about the influence of oxidative stress on MSC migration. We investigated the migratory capacity of adMSC in two different settings. Firstly, after preconditioning adMSC with H_2_O_2_ and GOx before induced migration (i.e., the start of the scratch assay, setting 1) or their continuous exposure during induced migration (setting 2). We found that both oxidative stress types had a stimulatory effect on adMSC migration and that preconditioning enhanced their capacity to migrate dose-dependently.

Furthermore, the migratory capacity of adMSC was improved by preconditioning with H_2_O_2_ rather than GOx. There is evidence that pre-incubation of bone marrow or Decidua Basalis MSC with H_2_O_2_ leads to enhanced migration when the migration process is induced without sustained oxidative stress [24,25]. Since the direct influences of H_2_O_2_ on the migration of MSC are sparsely studied, conclusions can be drawn from the light treatment of MSC with resulting ROS formation. Light treatment (low-level laser) of MSC induced increased intracellular ROS levels and was found to accelerate MSC’s migration through the extracellular signal-regulated kinases (ERK) 1/2 and focal adhesion kinase (FAK) pathways. Gingival fibroblasts have also been shown to migrate to a greater extent when treated with a blue diode laser. adMSC treated with light of different wavelengths have been shown to stimulate their migration capacities [51]. Considering this evidence and the limited information in the literature, the mechanisms underlying these migration-promoting effects in mesenchymal cells should be further investigated.

### 3.3. Differentiation of MSC under Oxidative Stress

The differentiation capacity of MSC is another important function in regeneration processes. In our study, all kinds of induced oxidative stress, even at very low concentrations of H_2_O_2_ or GOx, resulted in a concentration-dependent decrease in adipogenic and osteogenic differentiation in adMSC. Recent studies have described the influence of ROS on adipogenic or osteogenic differentiation of MSC through the activation or inhibition of the signaling cascade involved in the respective differentiation process [47,52]. Rodrigues et al. showed that ROS could induce the activation of mitogen-activated protein kinase (MAPK) pathways such as c-Jun N-terminal kinase (JNK) and p38MAPK, and ERK in MSC [53]. Atashi et al. (2015) concluded that ROS positively influence adipogenesis in MSC and other adipogenic progenitor cells, whereas osteogenesis is inhibited by increased amounts of ROS [47]. Osteogenic differentiation of bone marrow MSC is shown to be suppressed by oxidative stress upon exposure to H_2_O_2_ [26,54]. Oxidative stress is associated with many bone diseases, including osteoporosis, inflammatory joint disease, and impaired healing of fractures [55], and may, therefore, partly explain the development of these diseases.

However, a literature review on the adipogenic differentiation capacity of MSCs under oxidative stress reveals ambiguities, as some studies have reported reduced adipogenic differentiation, which may be related to oxidative stress-induced cell aging and senescence [56,57,58]. It remains unclear whether the restriction of MSC differentiation observed in our study, caused by both types of oxidative stress, is a controlled omission of differentiation or a targeted preservation of the stem cell status. The investigation of further differentiation pathways of oxidatively stressed MSC, e.g., differentiation into myofibroblasts, which can be induced under pro-inflammatory conditions [59], could shed light on this open question.

Ultimately, there is agreement that ROS and oxidative stress have an influence on MSC differentiation. It must be emphasized that all the results have arisen from differently designed in vitro models and the various models used to show apparent differences in their setup. For example, we deliberately omitted pyruvate from our experimental approaches, as the H_2_O_2_ is degraded more rapidly by this compound [60]. These differences in the culture methods, such as the composition of the cell culture medium, source of MSC, the timing of stimulation, level of oxidative stress, etc., will have an impact on many cellular dimensions and might explain the inconclusive data situation.

This study extends the current knowledge on the effects of long-term exposure of adMSC to pulsed H_2_O_2_ or sustained H_2_O_2_ exposure through the enzymatic reaction of GOx, as some differences between these two types of treatment were revealed:

1. Despite nearly equal maximum H_2_O_2_ concentrations, direct pulsed exposure of adMSC to H_2_O_2_ resulted in significantly higher ROS development compared to sustained GOx-induced H_2_O_2_ exposure.
2. Direct pulsed exposure of adMSC to H_2_O_2_ showed higher cytotoxicity than sustained GOx-induced H_2_O_2_ exposure.
3. Antioxidant enzymes were more abundant during sustained GOx-induced H_2_O_2_ exposure.
4. Release of inflammatory factors was higher during pulsed H_2_O_2_ exposure.
5. Migratory activity of adMSC was increased by exposure to H_2_O_2_ before migration and showed high sensitivity to the degree of oxidative stress since exposure to higher but non-cytotoxic pulsed H_2_O_2_ concentrations during migration completely blocked their migratory activity.
6. Both adipogenic and osteogenic differentiation is reduced under all H_2_O_2_ exposure conditions.

These results indicate that the direct pulsed H_2_O_2_ exposure induces higher oxidative stress in adMSC in vitro than in GOx-induced, sustained H_2_O_2_ exposure. This is probably triggered by a better cellular adaptation to the oxidative stress conditions when they are constant. However, patient-specific characteristics, such as age, gender, chronic diseases, and genetic variants might influence the resistance of MSC to oxidative stress [61,62]. These individual characteristics could impact not only the results shown in this study but also the effectiveness of cell therapy. Therefore, further studies are needed to understand the mechanisms underlying the regulation of these functions. Nevertheless, these results are another step towards a broader knowledge of the effects of oxidative stress on adMSC.

## 4. Materials and Methods

### 4.1. adMSC Isolation and Cell Culture

Primary human adipose tissue-derived mesenchymal stem/stromal cells (adMSC) were isolated from liposuction tissue of healthy patients with an average age of 50 ± 10 years. The aspirated tissue was transported overnight at room temperature (RT), and the stromal vascular fraction (SVF) was isolated according to a previously described protocol [63]. CD34-positive subpopulation was isolated from SVF based on a standard protocol [64], and CD34-positive cells were seeded in passage 1 with 12 mL of DMEM (Gibco by Life technologies, Paisley, UK) containing 10% FCS (PAN Biotech, Aidenbach, Germany), 1% P/S, and 0.4% GlutaMax^TM^ (Gibco, by Life technologies, Paisley, UK), hence referred to as the ‘complete culture medium’ and cryopreserved in passage 2 based on the previously described procedure [65]. For all cell culture experiments, adMSC were thawed from passage 2 with a standard laboratory protocol and seeded in a 75 cm^2^ flask (Greiner Bio-one, Frickenhausen, Germany) [66]. All experiments were carried out in passage 4. Unless otherwise stated, cells were seeded at a density of 20,000 cells/cm^2^ in plates 4- to 96-well (Greiner Bio-one, Frickenhausen, Germany). DNA staining with DAPI (Sigma Aldrich, Saint Louis, MO, USA) microscopically confirmed the absence of mycoplasma contamination. 

### 4.2. Treatment of adMSC with Oxidative Stressors, H_2_O_2_, and GOx 

After 72 h of cell cultivation in a complete culture medium without pyruvate, cells were supplemented with H_2_O_2_ (30% *w*/*w* solution, Sigma Aldrich, Saint Louis, MO, USA) or GOx (Sigma Aldrich, Saint Louis, MO, USA) with a final concentration of 0.01, 0.05, 0.1, 0.25 mM and 0.1, 0.5, 1, 2.5, and 5 mU/mL, respectively. Unless otherwise stated, these were the treatment media used for the experiments. adMSC with media without these oxidative stressors will be referred to as control hereafter. Treatments were repeated every 2–3 days throughout the experiment. 

### 4.3. Extracellular H_2_O_2_ and Intracellular ROS

The amount of H_2_O_2_ in the treated media was quantified using Amplex UltraRed (Thermo Fischer Scientific, Waltham, MA, USA) according to the manufacturer’s instructions. Complete media treated with different concentrations of H_2_O_2_ and GOx were measured for 1 h to 48 h. Relative fluorescent intensity was measured using a microplate reader (TECAN, Männedorf, Switzerland). The H_2_O_2_ concentration was calculated using a standard curve. 

Intracellular ROS were detected using 2′-7′-dichlorofluorescein diacetate (CM-H_2_DCFDA, Thermo Fischer Scientific, Waltham, MA, USA) ROS indicator. 72 h after seeding, CM-H_2_DCFDA was added to the cells as a probe at a final concentration of 10 µM in pre-warmed phosphate-buffered saline (PBS, PAN Biotech, Aidenbach, Germany) without Ca^2+^ and Mg^2+^ and incubated at 37 °C for 30 min. After incubation, the fluorescent probe was removed, and the cells were washed with 1 × PBS without Ca^2+^ and Mg^2+^. Finally, cells were exposed to different concentrations of H_2_O_2_ and GOx, as described above, whereas cell cultures without treatment served as controls. Intracellular ROS accumulation was measured using a microplate reader (TECAN, Männedorf, Switzerland). Fluorescence was quantified at 492 nm excitation and 527 nm emission every 10 min for 50 min until saturation was reached. The results were normalized to the cell count to estimate the amount of accumulated ROS per individual cell. 

### 4.4. Cell Number 

Quantification of cell number was conducted with Hoechst H33342 (Applichem, Darmstadt, Germany). Cells were first washed with PBS without Ca^2+^ and Mg^2+^ and fixed with 4% paraformaldehyde (PFA, Sigma-Aldrich, Saint Louis, MO, USA). Cells were washed three times with PBS without Ca^2+^ and Mg^2+^ and incubated with 5 µg/mL Hoechst H33342 for 10 min in the dark, and the washing step was repeated. Images were acquired using the Hermes WiScan system (IDEA Bio-Medical, Rehovot, Israel) and analyzed using the Athena software (IDEA Bio-Medical, Rehovot, Israel). The total number of nuclei and the average and standard deviation of triplicates for each treatment were calculated for the cell number. This process was also performed on days 1, 3, and 7. 

### 4.5. Metabolic Activity 

The metabolic activity of adMSC was determined with the CellTiter 96 Aqueous One Solution Cell Proliferation Assay (MTS, Promega, Madison, WI, USA) on days 1, 3, and 7 after treatment, according to the manufacturer’s instructions. For this purpose, the cells were incubated with the MTS solution for 1.5 h, using the MTS solution without cells as a blank. Afterward, the supernatant and the blank were transferred into an ELISA reading plate (Greiner Bio-one, Frickenhausen, Germany), and the absorbance was measured in a microplate reader (TECAN, Männedorf, Switzerland) at an absorbance wavelength of 490 nm (reference wavelength 620 nm). 

### 4.6. Cell Stress-Related Proteins 

#### 4.6.1. Cell Stress 

26 cell stress-related proteins (Table S1) were detected using their respective antibodies coated on the membrane from human cell stress array kit (R & D Systems, Minneapolis, MN, USA). For this, cells were treated with 0.05, 0.1 mM H_2_O_2_, and 2.5 mU/mL GOx for 24 and 48 h. The cell supernatants were then collected and stored at −80 °C for further cytokines analyses. Whereas cells were lysed for 30 min at 4 °C with a lysis buffer containing a cocktail of protease inhibitors (10 μg/mL Aprotinin, Leupeptin, and Pepstatin, respectively; all from R & D System, Minneapolis, MN, USA). The lysates were centrifuged at 14,000× *g* for 5 min at 4 °C. For each analysis, 250 µg of protein was pooled from the lysates of 5 individuals. Cell stress-related proteins were detected using the kit according to the manufacturer’s instructions. For this, chemiluminescence blots were recorded with ChemiDoc XRS (Bio-Rad Laboratories GmbH, Munich, Germany), and protein intensity was quantified by densitometric analysis of the blot using the Image Lab 3.0.1 software (Bio-Rad, Laboratories GmbH, Munich, Germany).

#### 4.6.2. Proinflammatory Cytokines

For the determination of pro-inflammatory cytokines, IL-8 ELISA and NF-ҡB translocation were performed.

For the IL-8, the previously processed cell supernatants were used and analyzed with the ELISA (R&D System, Minneapolis, MN, USA) according to the manufacturer’s instructions. The measured IL-8 concentration in pg/mL was normalized to the concentration of the lysates for each treatment. 

NF-ҡB nuclear translocation was determined after 8 and 24 h treatment with the same concentrations of H_2_O_2_ (0.05, 0.1 mM) and GOx (2.5 mU/mL). As a positive control, adMSC incubated with 50 ng/mL TNFα (Proteintech, Rosemont, IL, USA) for 30 min was included. Cells were then fixed with 4% PFA, and NF-ҡB antibody staining (Cell Signaling, Danvers, MA, USA) was performed according to the manufacturer’s instructions. The cell nuclei were stained with Hoechst H33342, as previously described. After staining, imaging was performed using the Hermes WiScan microscope (IDEA Bio-Medical, Rehovot, Israel). NF-ҡB translocation was quantified by the fluorescence intensity of the nuclei using Athena software (IDEA Bio-Medical, Rehovot, Israel). The average intensity of the nuclei was calculated from the sum of all mean intensities divided by the number of nuclei measured. 

#### 4.6.3. Adipokines

The previously processed cell supernatants were used for the detection of 58 different obesity-related adipokines (Table S2) with a Proteome Profiler Human Adipokine Array Kit (R & D Systems, Minneapolis, MN, USA). 500 µL of supernatants were pooled from 5 individuals. Adipokines were detected using the kit according to the manufacturer’s instructions. For this, chemiluminescence blots were recorded with ChemiDoc XRS (Bio-Rad Laboratories GmbH, Munich, Germany), and the protein intensity was quantified by densitometric analysis of the blot using the Image Lab 3.0.1 software. 

### 4.7. Intracellular Basal Calcium Levels

The intracellular basal Ca^2+^ levels were determined via flow cytometry by staining the cells with 5 µM Fluo-3-acetoxymethyl ester AM (fluo-3/AM) calcium indicator (Life Technologies Corporation, Eugene, OR, USA). For this approach, cells were first seeded in a 24-well plate and treated with 0.01, 0.05, 0.1, 0.25 mM H_2_O_2_ and 0.1, 0.5, 1, 2.5, and 5 mU/mL GOx for 24 h. After treatment with the stressors, the cells were washed, trypsinized, and the cell suspension was recovered in the FACS tube (BD Biosciences, Franklin Lakes, NJ, USA). The intracellular basal Ca^2+^ level was determined according to a previously published protocol [67]. CellQuest Pro 4.0.1 software (BD Biosciences, Franklin Lakes, NJ, USA) was used for data acquisition and analyses of the results.

### 4.8. Determination of Cell Migration

Cell migration was investigated with a scratch assay. Cells were seeded at 29,000 cells/cm^2^ in a 96-well plate to achieve complete cell spreading and an almost 100% confluent monolayer. 24 h after seeding, the medium was replaced with a complete culture medium, without phenol red diluted with a red cell tracking dye, DMSO free (Abcam, Cambridge, UK) according to the manufacturer’s instructions. The cells were divided into two settings according to their experimental arrangement. 72 h after seeding, the cells were incubated with 0.01, 0.05, and 0.1 mM H_2_O_2_ and 0.1, 0.5, 1, and 2.5 mU/mL GOx.

The first settings consisted of cells preconditioned for 24 h with H_2_O_2_ and GOx. Afterward, the confluent monolayer was scratched with an Incucyte^®^ Woundmaker (Sartorius, Göttingen, Germany). The cells were washed three times with PBS without Ca^2+^ and Mg^2+^, and the media were replaced with supplemented low serum media (1% FCS) without pyruvate to limit the proliferation factor. Live-cell imaging was observed immediately after scratch using the Hermes WiScan system (IDEA Bio-Medical, Rehovot, Israel) at 10-fold magnification in a humidified atmosphere with 37 °C and 5% CO_2_ for 20 h. Quantification of the wound area was performed using Fiji ImageJ software version 1.8.0_72 [68], and the percentage of wound closure was calculated by the following formula:

*(initial wound area − final wound area)/initial wound area ×* 100%.

The second setting was 2 h preconditioned adMSC and 15 h in treatment. The cells were incubated with the same concentrations of H_2_O_2_ and GOx described above for 2 h. Subsequently, the scratch was made, and the cells were treated again with the same concentrations of H_2_O_2_ and GOx in the supplemented media, with 10% FCS without pyruvate. Imaging was performed immediately after the scratch and continued for 15 h. 

### 4.9. Differentiation of the adMSC

#### 4.9.1. Quantification of Cell Number

The cell number of adipogenically differentiated cells was determined 14 days after stimulation and of osteogenically differentiated cells, 14, 21, and 28 days after stimulation. Quantification of cell number was performed using crystal violet staining, as previously described [64].

#### 4.9.2. Quantification of Metabolic Activity

In addition, an MTS assay was conducted on the days mentioned above to check the metabolic activity of the cells and was carried out as previously described.

#### 4.9.3. Adipogenic Differentiation 

To observe the effect of oxidative stress on adipogenic differentiation, cells were treated with 0.01, 0.05, and 0.1 mM H_2_O_2_ and 0.1, 0.5, 1, 2.5, and 5 mU/mL GOx. The procedure of adipogenic differentiation was performed based on a previously published protocol [69]. The cells were divided into two groups, the first group being the unstimulated (US) adMSC, in which the above-defined concentrations of H_2_O_2_ and GOx were added to the medium. In contrast, cells without treatment served as controls (US control). The second group was the adipogenically stimulated (AS) adMSC, in which the adipogenic media (containing 1 µM dexamethasone, 500 µM 3-isobutyl-1-methylxanthine, 200 µM indomethacin, and 10 µM insulin; all from Sigma-Aldrich, Saint Louis, MO, USA) was added to the cells. At the same time, they were simultaneously treated with the above-defined concentrations of H_2_O_2_ and GOx, and the control cells (AS control) without treatment. After 14 days of stimulation and treatment, adipogenic differentiation was determined by Bodipy staining, as previously described [69].

#### 4.9.4. Osteogenic Differentiation

The osteogenic differentiation capacity was determined by quantifying the enzyme alkaline phosphatase (ALP) activity. Stimulation of osteogenesis was realized by replacing the medium every 2–3 days and simultaneous repeated treatment with the stressors. The cells were divided into two groups, the first group being the unstimulated (US) adMSC, in which the above-defined concentrations of H_2_O_2_ and GOx were added to the medium. Cells without treatment served as controls (US control). The second group was the osteogenically stimulated (OS) adMSC, in which the osteogenic media (containing 0.25 g/L, ascorbic acid, 1 µM dexamethasone, and 10 mM β-glycerolphosphate; all from Sigma-Aldrich Saint Louis, MO, USA) was added to the cells. At the same time, they were simultaneously treated with the above-defined concentrations of H_2_O_2_ and GOx, and the control cells (OS control) without treatment. ALP quantification was carried out according to a previously established protocol [69] and was performed after 14, 21, and 28 days of stimulation and treatment. For visual analysis, cells were stained with a solution of 67 mM 2-Amino-2-methyl-1.3-propanediol, 2.7 mM Naphthol AS-MX phosphate, and 2.7 mM Fast Red Violet LB Salt (all from Sigma Aldrich, Saint Louis, MO, USA) in H_2_O according to a protocol published [69], and imaged using a Zeiss Microscope (Zeiss, Oberkochen, Germany).

### 4.10. Data and Statistical Analysis

All experiments were performed independently on cells from five or more different donors, each in triplicates, and the mean of each triplicate was used for one individual. Data were visualized and statistically analyzed with Microsoft Excel 2010 (Microsoft, Redmond, WA) and GraphPad Prism, Version 6.05 (GraphPad Software Inc., San Diego, CA, USA). Data were presented as bars with standard deviations and boxplots. The horizontal line within the box plot indicates the median, with whiskers showing the minimum and maximum data points. The statistical significance of the data set was calculated using a two-way analysis of variance, followed by Dunnett’s multiple comparison tests, and the level of significance was set to a *p*-value of 0.05.

### 4.11. Ethical Statement

All tissue samples analyzed for this experiment were collected with the patient’s consent. The use of the tissue samples was approved by the local ethics committee under registration A2019-0107. 

## Data Availability

The data presented in this study are available in the article and supplementary materials.

## Supplementary Materials

The supporting information can be downloaded at: https://www.mdpi.com/article/10.3390/ijms232113435/s1.
