# Supplementary material for: Oxidative Stress Response in Adipose Tissue-Derived Mesenchymal Stem/Stromal Cells
Source: Int J Mol Sci. 2022 Nov 3;23(21):13435. doi: 10.3390/ijms232113435 (PMC9654835; doi:10.3390/ijms232113435)
Supplement: Supplementary file 1 [file ijms-23-13435-s001.zip › ijms-1996107-supplementary.pdf]

Table S1. Coordinates of the spots and list of the 26 different stress proteins on the Human Cell Stress Array membrane.

Coordinate	Analyte/Control	Coordinate	Analyte/Control
A1, A2	Reference Spots	C11, C12	IDO
A21, A22	Reference Spots	C13, C14	Phospho-JNK Pan (T183/Y185)
B3, B4	ADAMTS1	C15, C16	NFκB1
B5, B6	Bcl-2	C17, C18	p21/CIP1
B7, B8	Carbonic Anhydrase	C19, C20	p27
B9, B10	Cited-2	D3, D4	Phospho-p38α (T180/Y182)
B11, B12	COX-2	D5, D6	Phospho-p53 (S46)
B13, B14	Cytochrome c	D7, D8	PON
B15, B16	Dkk-4	D9, D10	PON2
B17, B18	FABP-1	D11, D12	PON3
B19, B20	HIF-1α	D13, D14	Thioredoxin-1
C3, C4	HIF-2α	D15, D16	Sirtuin2/SIRT2
C5, C6	Phospho-HSP27 (S78/S82)	D17, D18	Superoxide dismutase2/SOD2
C7, C8	HSP60	D19, D20	Negative Control
C9, C10	HSP70	E1, E2	Reference Spots

Table S2. Coordinates of the spots and list of the 58 different adipokines on the Human Adipokines Array membrane.

Coordinate	Analyte/Control	Coordinate	Analyte/Control
A1, A2	Reference Spots	C19, C20	IL-6
A5, A6	Adiponectin/Acrp30	C21, C22	CXCL8/IL-8
A7, A8	Angiopoietin-1	C23, C24	IL-10
A9, A10	Angiopoietin-2	D1, D2	IL-11
A11, A12	Angiopoietin-like 2	D3, D4	LAP (TGF-β1)
A13, A14	Angiopoietin-like 3	D5, D6	Leptin
A15, A16	BAFF/BLyS/TNFSF13B	D7, D8	LIF
A17, A18	BMP-4	D9, D10	Lipocalin-2/NGAL
A19, A20	Cathepsin D	D11, D12	CCL2/MCP-1
A23, A24	Reference Spots	D13, D14	M-CSF
B1, B2	Cathepsin L	D15, D16	MIF
B3, B4	Cathepsin S	D17, D18	Myeloperoxidase
B5, B6	Chemerin	D19, D20	Nidogen-1/Entactin
B7, B8	Complement Factor D	D21, D22	Oncostatin M (OSM
B9, B10	C-Reactive Protein/CRP	D23, D24	Pappalysin-1/PAPP-A
B11, B12	DPPIV/CD26	E1, E2	PBEF/Visfatin
B13, B14	Endocan	E3, E4	Pentraxin-3/SG-14
B15, B16	EN-RAGE	E5, E6	Pref-1/DLK-1/FA1
B17, B18	Fetuin B	E7, E8	ProproteinConvertase9/PCSK9
B19, B20	FGF basic	E9, E10	RAGE
B21, B22	FGF-19	E11, E12	CCL5/RANTES
B23, B24	Fibrinogen	E13, E14	Resistin
C1, C2	Growth Hormone	E15, E16	Serpin A8/AGT
C3, C4	HGF	E17, E18	Serpin A12
C5, C6	ICAM-I/CD54	E19, E20	Serpin E1/PAI-1
C7, C8	IGFBP-2	E21, E22	TIMP-1
C9, C10	IGFBP-3	E23, E24	TIMP-3
C11, C12	IGFBP-4	F1, F2	Reference Spots
C13, C14	IGFBP-6	F5, F6	TNF-α
C15, C16	IGFBP-rp1/IGFBP-7	F7, F8	VEGF
C17, C18	IL-1β/IL-1F2	F23, F24	Negative Controls

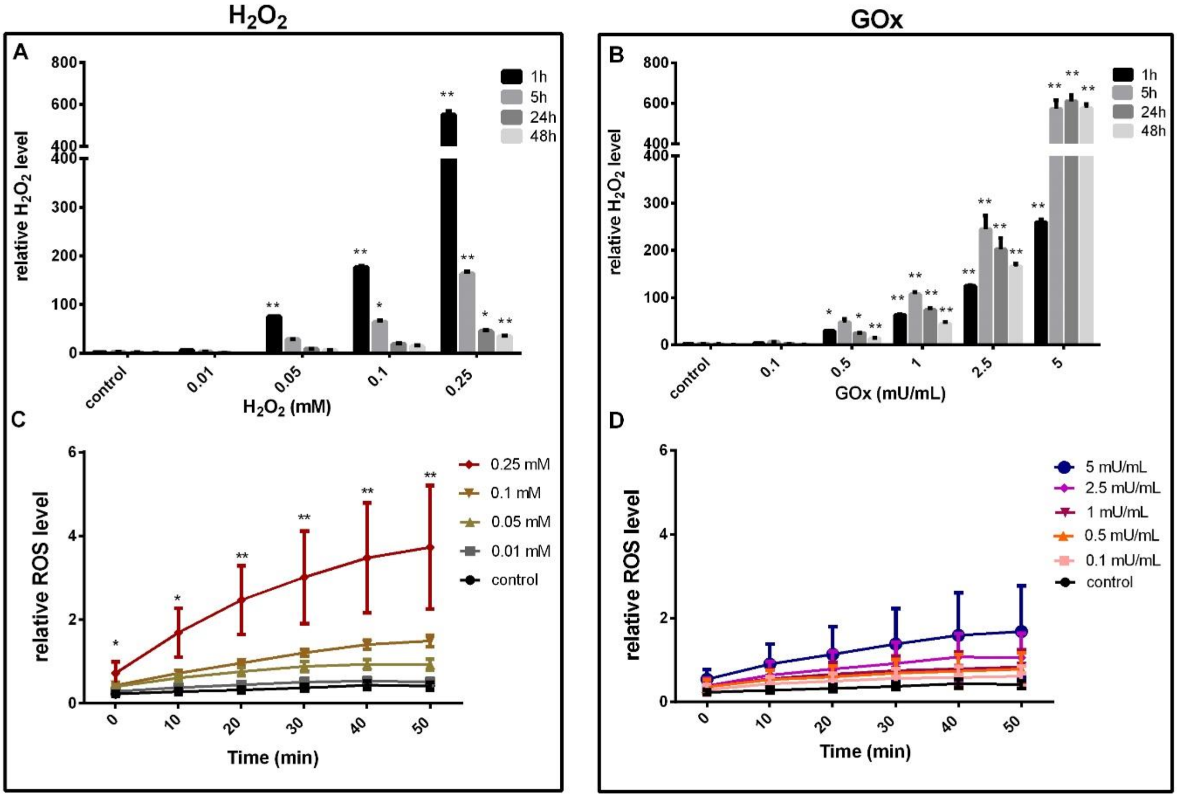

Figure S1. Quantification of H_2O_2 in the cell culture medium and analysis of formation of intracellular ROS. (A) Quantification of H_2O_2 in the cell culture medium treated with different concentrations of H_2O_2 and (B) GOx for 1, 5, 24 and 48 h. (C) Intracellular ROS levels quantified after treatment with different concentrations of H_2O_2 and; (D) GOx for 50 min (data presented as bar graphs for H_2O_2 concentration measured from the fluorescent intensity with Amplex UltraRed, and ROS measured from relative fluorescent intensity normalized to cell count with CM-H_2DCFDA ROS indicator; n = 5, mean and standard deviation of the mean; significant data compared to the respective controls; 2-way ANOVA with Dunnett's multiple comparison test, *$p < 0.05$, **$p < 0.001$).

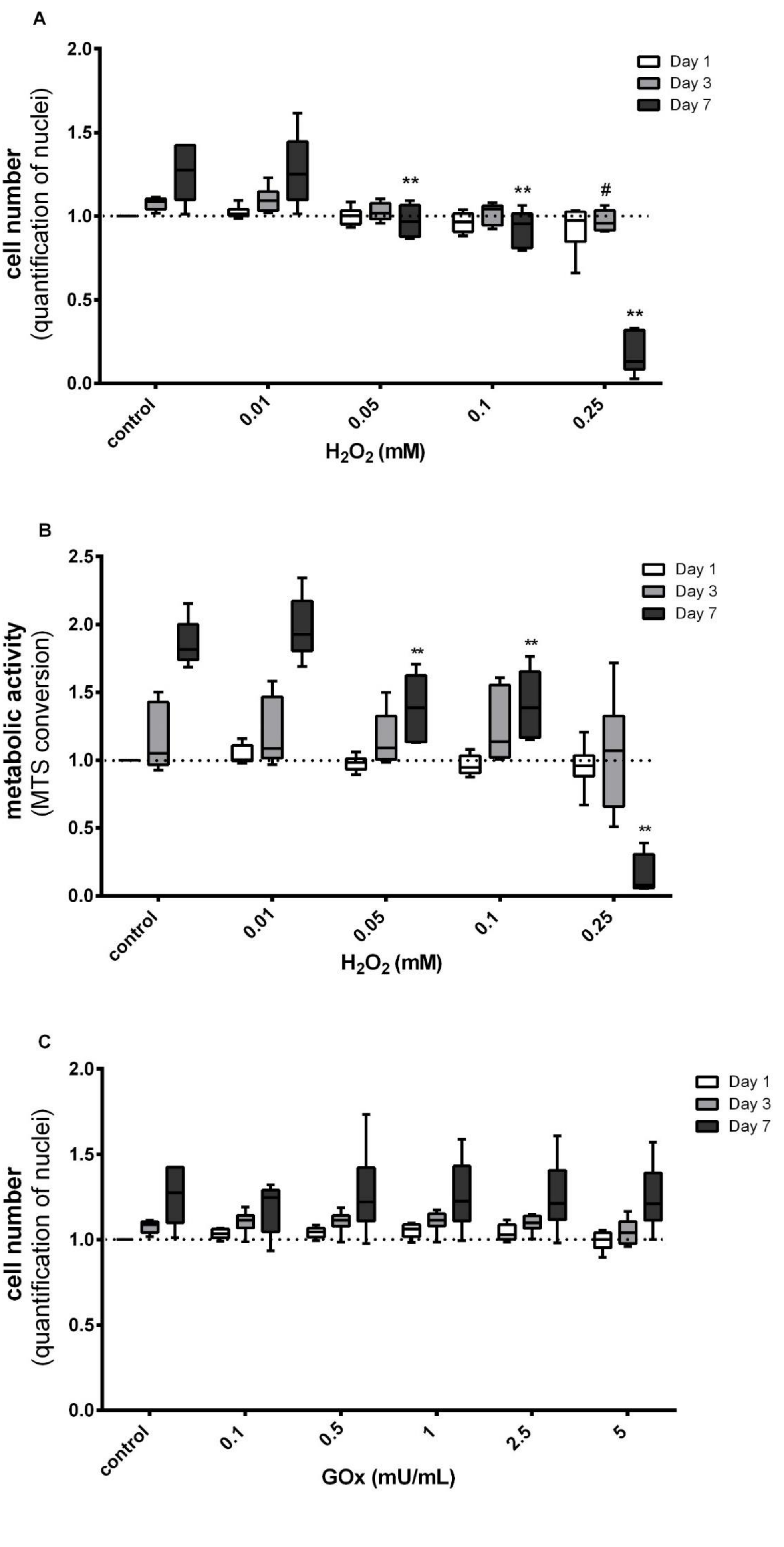
A
cell number
(quantification of nuclei)
2.0
1.5
1.0
0.5
0.0
Day 1
Day 3
Day 7
**
**
#
**
control
0.01
0.05
0.1
0.25
H2O2 (mM)
B
metabolic activity
(MTS conversion)
2.5
2.0
1.5
1.0
0.5
0.0
Day 1
Day 3
Day 7
**
**
**
control
0.01
0.05
0.1
0.25
H2O2 (mM)
C
cell number
(quantification of nuclei)
2.0
1.5
1.0
0.5
0.0
Day 1
Day 3
Day 7
control
0.1
0.5
1
2.5
5
GOx (mU/mL)

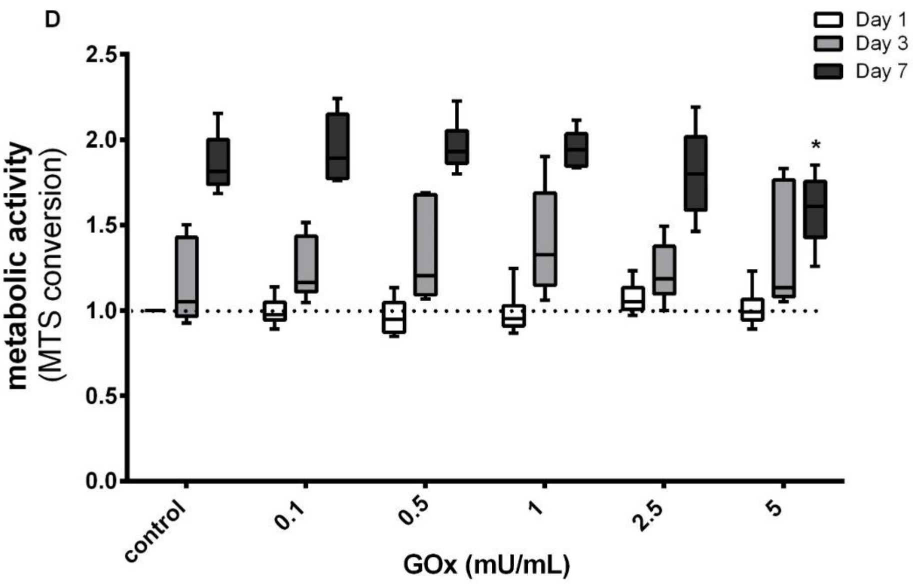

Figure S2. The effect of H_2O_2 on (A) cell numbers and (B) metabolic activity; and the effect of GOX on (C) cell number and (D) metabolic activity after day 1, 3 and 7 of repeated treatment ((**A**): cell number analysed by quantification of nuclei upon staining with Hoechst H33342, (**B**): metabolic activity quantified by MTS conversion assay). The data set was normalized compared to the control on day 1 after the treatment; (n = 6, # * significant data compared to the control group on the respective days of the treatment; 2-way ANOVA with Dunnett's multiple comparison test, $^{\#}$ $p < 0.05$, *$p < 0.05$, **$p < 0.001$).

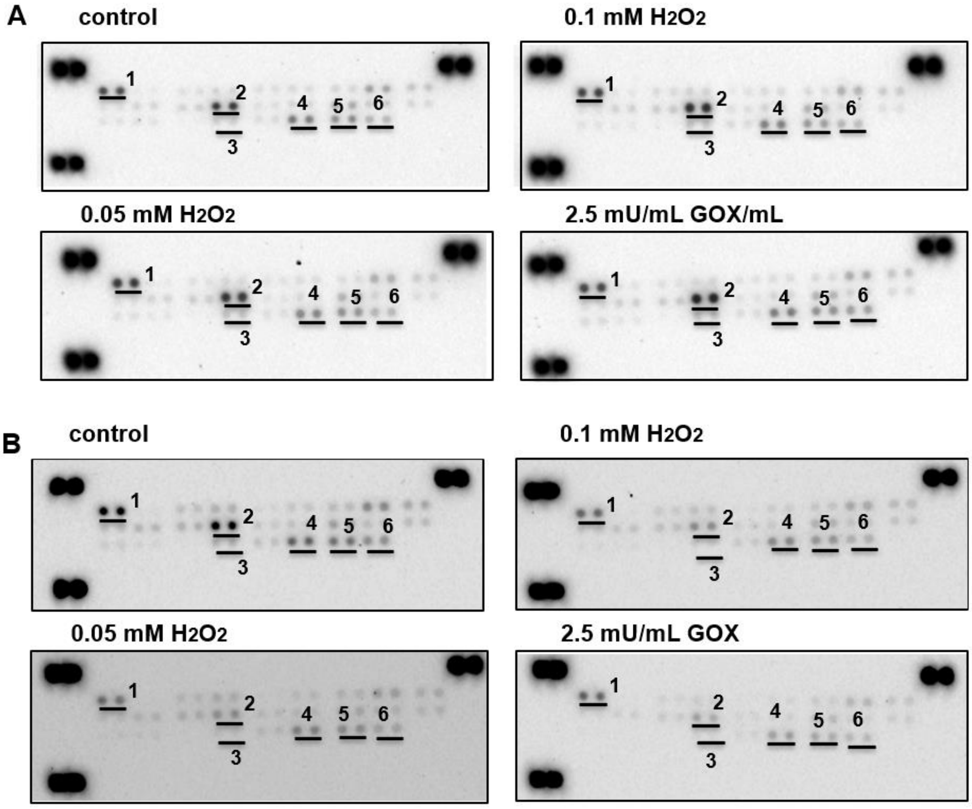

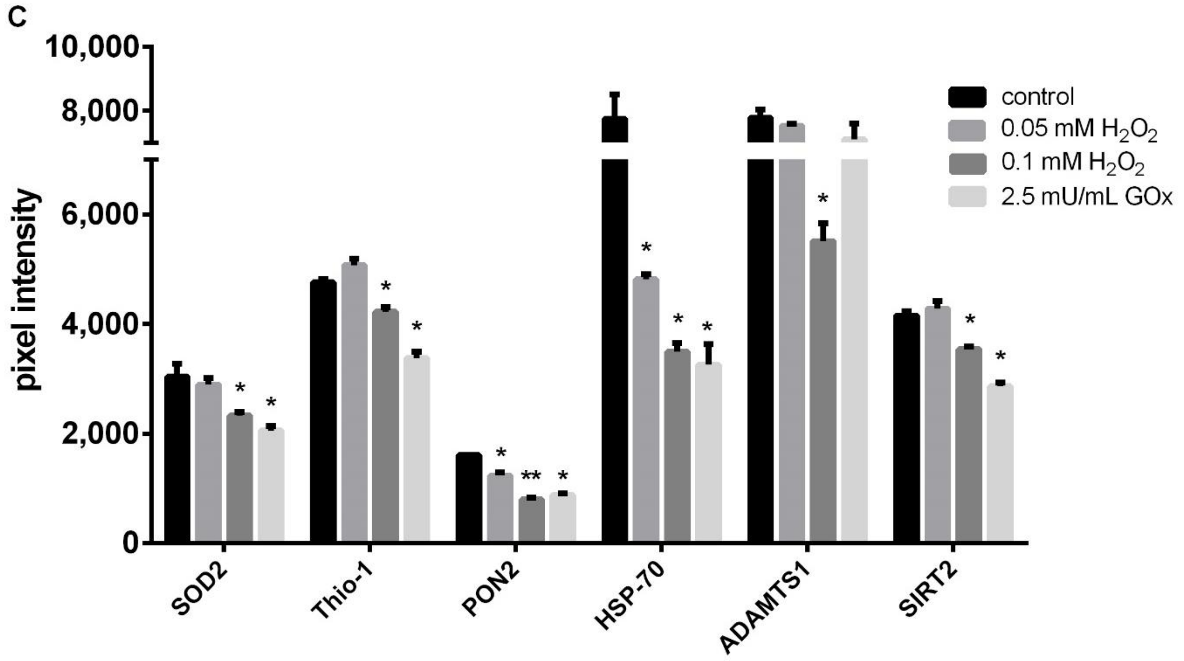

Figure S3. Analysis of human cell stress proteins. adMSC was treated with 0.05 and 0.1 mM H_2O_2, or 2.5 mU/mL GOx respectively. adMSC lysates were analyzed with 'Proteome Profiler Human Cell Stress Array Kit' (A) 24 h and (B) 48 h after treatment; protein spots on the membrane, each duplicate represents (1) ADAMTS1 (2) HSP-70 (3) PON2 (4) Thio-1 (5) Sirt2 (6) SOD2. (C) Pixel intensity of the proteins quantified (protein intensity was quantified by densitometry analysis of the spots using the Image Lab 3.0.1 software; n=5, significant data compared to the control; 2-way ANOVA with Dunnett's multiple comparison test, *$p < 0.05$, **$p < 0.001$).

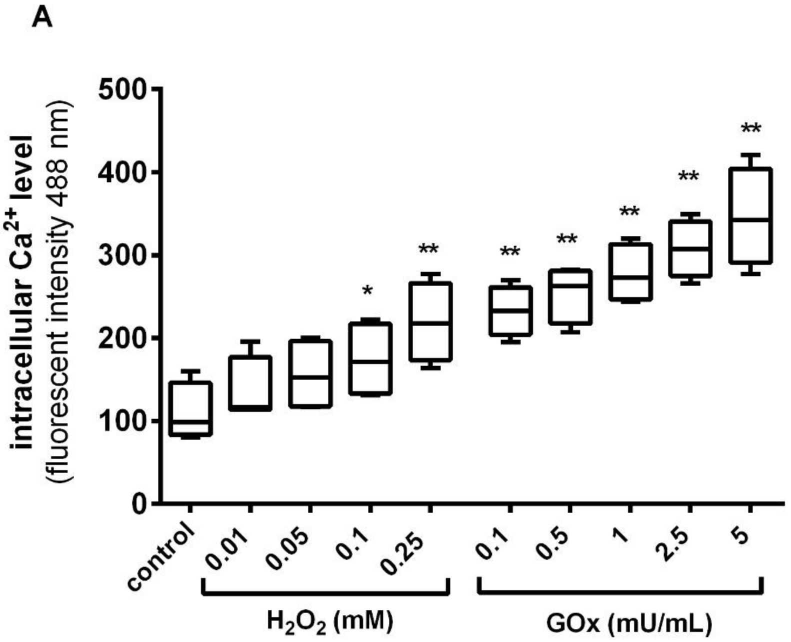

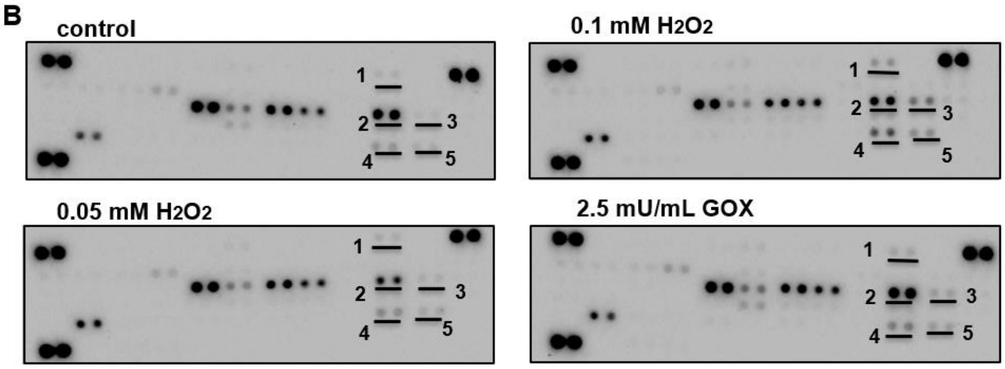

Figure S4. Inflammation related signaling upon treatment of adMSC with different concentrations of H_2O_2, and GOx. (A) Intracellular *Ca*$^{2+}$ level detection with the fluo-3/AM calcium indicator (analysis by flow cytometry). (B) Depiction of differentially released adipokines with 'Human Adipokine Array kit'. Protein spots on the membrane, each duplicate represents (1) CTSD (2) IL-6 (3) IL-8 (4) PAPP-A (5) TIMP-1 (n = 5, significant data compared to the control; 2-way ANOVA with Dunnett's multiple comparison test, *$p < 0.05$, **$p < 0.001$).
